# Prone position failure in moderate-severe acute respiratory distress syndrome: and now?

**DOI:** 10.5935/2965-2774.2023.Edit-1.v35n2-en

**Published:** 2023

**Authors:** Carmen Silvia Valente Barbas, Corinne Taniguchi

**Affiliations:** 1 Adult Intensive Care Unit, Hospital Israelita Albert Einstein - São Paulo (SP), Brazil; 2 Physiotherapist - São Paulo (SP), Brazil

In 2013, a randomized, control, prospective, multicenter PROSEVA study was published^(1)^ and compared 16 hours early prone to supine position in 474 acute respiratory distress syndrome (ARDS) patients with a partial pressure of oxygen/fraction of inspired oxygen (PaO_2_/FiO_2_) < 150 and with a positive end-expiratory pressure (PEEP) > 5cmH_2_O, and the study revealed that the prone position in these patients decreased the 28-day and 90-day mortality rates and that the prone position is indicated in these cases. This study also showed that the PaO_2_/FiO_2_ ratio was higher at Days 3 and 5 in the prone group than in the supine group. The plateau pressure of the respiratory system (Pplat_rs_) was 2cmH_2_O lower in the Prone Group than in the Supine Group at Day 3. The mean rate of prone positioning per patient was 4 ± 4, and the mean duration per session was 17 ± 3 hours. Neuromuscular blockers were used for 5.6 ± 5.0 days in the Supine Group and 5.7 ± 4.7 days in the Prone Group (p = 0.74), and intravenous sedation was given for 9.5 ± 6.8 and 10.1 ± 7.2 days in the two groups, respectively (p = 0.35). The authors stratified the patients according to quartiles of the PaO_2_/FIO_2_ ratio at enrollment, and they did not find differences in the outcomes. The rate of successful extubation was significantly higher in the prone group. However, the duration of invasive mechanical ventilation (MV), length of stay in the intensive care unit (ICU), incidence of pneumothorax, rate of use of noninvasive ventilation after extubation, and tracheotomy rate did not differ significantly between the two groups.^(1)^

In 2020, Lee et al.^(2)^ reported 116 ARDS patients who received prone position ventilation, of whom 45 (38,8%) were ICU survivors. Although there was no difference in the PaO_2_/FIO_2_ ratio before the first prone session between ICU survivors and nonsurvivors, ICU survivors had a higher PaO_2_/FIO_2_ ratio after prone positioning than nonsurvivors, with a significant between-group difference (p < 0.001). In the multivariate Cox regression analysis, prone responders (hazard ratio - HR 0.11; 95% confidence interval - 95%CI 0.05 - 0.25), immunocompromised conditions (HR 2.15; 95%CI 1.15 - 4.03), and Sequential Organ Failure Assessment score (HR 1.16; 95%CI 1.06 - 1.27) were significantly associated with 28-day mortality. In this study, improvement in oxygenation after the first prone positioning was a significant predictor of survival in patients with moderate-to-severe acute respiratory distress syndrome.

In another study, Jochmans et al.^(3)^ analyzed 103 patients (95% ARDS) during 231 prone sessions with a mean length of 21.5 ± 5 hours per session. They presented a significant increase in pH, static compliance and PaO_2_/FiO_2_ with a significant decrease in partial pressure of carbon dioxide (PaCO_2_), Pplat_rs_, phase 3 slope of the volumetric capnography, partial pressure of end tidal carbon dioxide (P_et_CO_2_), physiological dead space ventilation (V_D_/V_T-phy_) and ∆P (driving pressure). The beneficial physiological effects continued after 16 hours of prone positioning and at least up to 24 hours in some patients. In the evolution of the respiratory parameters during the first session and during the pooled sessions, there were no predictors of response to the prone position that were found, whether before, during or 2 hours after the return to the supine position.

In ARDS patients, the change from supine to prone position generates a more even distribution of the gas-tissue ratios along the dependent-nondependent axis and a more homogeneous distribution of lung stress and strain.^(4)^ The change to the prone position is generally accompanied by a marked improvement in arterial blood gases, which is mainly due to better overall ventilation/perfusion matching. Improvement in oxygenation and reduction in mortality are the main reasons to implement the prone position in patients with ARDS.^(4)^

Recently, prone position ventilation has been widely used in acute respiratory failure due to the coronavirus disease 2019 (COVID-19) pandemic. Kharat et al.^(5)^ reviewed 24 observational studies of COVID-19 ARDS patients who received prone position ventilation. Three studies compared COVID-19-related ARDS patients placed in the prone position to patients who were not placed in the prone position. The mortality was not significantly different between the groups [odds ratio - OR 0.45 (0.09 - 2.18)], but the heterogeneity was extremely high (*I*^2^ = 91%). Fifteen studies had PaO_2_/FIO_2_ data available before and during proning. Except in two studies, the mean increase in the PaO_2_/FIO_2_ ratio in the prone position was more than 20mmHg from its value before proning, a common threshold used to define responders. The rate of responders ranged from 9 to 77%. Seven studies provided data on static compliance of the respiratory system in supine preprone and prone patients. It significantly increased after a few hours in the prone position by 2mL/cmH_2_O on average (*z* = -2.68; p < 0.01); *I*^2^ = 30%). The short-term physiological response is consistent with what is known in classic non-COVID-19 ARDS. Three studies in intubated COVID-19 patients found that the outcome was better in responders than in nonresponders [OR 0.44 (0.27 - 0.71), p < 0.01] without any heterogeneity (*I*^2^ = 0%). There are currently no randomized study of the prone position in COVID-19 ARDS patients.

Fossali et al.^(6)^ studied the lung protective effects of the prone position in 21 patients with COVID-19-ARDS by analyzing computed thoracic tomography and electrical impedance tomography. They observed that the prone position induced extensive alveolar recruitment in the dorsal regions and alveolar derecruitment in the ventral lung regions. Dorsal recruitment reduces the risk of regional atelectrauma in comparison to the supine position. They observed that ventral lung regions, after pronation, are characterized by a decreased fraction of ventilated nonperfused units and a reduced dead space/shunt ratio. Dead space measured by electrical impedance tomography was reduced in the ventral regions of the lungs, and the dead-space/shunt ratio decreased significantly (5.1 [2.3 - 23.4] *versus* 4.3 [0.7 - 6.8]; p = 0.035), showing an improvement in ventilation-perfusion matching.

In this issue of Critical Care Science, Sanabria-Rodríguez et al.^(7)^ analyzed 724 patients with COVID-19 and severe ARDS who received invasive MV and who, due to refractory hypoxemia, underwent prone positioning. One hundred fifty-nine patients (21.9%) did not respond to pronation. The median PaO_2_/FIO_2_ variation was 62.8% in responders (interquartile range - IQR 42.85 - 100) and 2.7% (IQR 7.63 - 11.36) in nonresponders. Nonresponders had higher D-dimer levels and prepronation PaO_2_/FIO_2_ ratios, more frequent lung consolidation, more frequent need for 3 or more sedatives, and a longer time between the start of MV and the start of pronation. The PaO_2_/FIO_2_ response after prone positioning was lower when the preintubation or prepronation PaO_2_/FIO_2_ was higher, when the driving pressure was ≥ 15cmH_2_O and in patients who received more sedatives. The logistic regression model showed that the chance of nonresponding to prone positioning increased significantly each day after the start of invasive MV. The model also showed that the likelihood of nonresponding was higher with a lung consolidation or mixed radiological pattern than with a ground-glass pattern. The authors observed a low correlation between preintubation PaO_2_/FIO_2_ and prepronation PaO_2_/FIO_2_. The likelihood of nonresponding to prone positioning was lower in patients with a preintubation PaO_2_/FIO_2_ of 100 - 150 than in patients with a preintubation PaO_2_/FIO_2_ of 150. Assessment of discrimination capacity showed that the model correctly predicted nonresponse to prone positioning in 79.28% of cases, with a proper discrimination capacity (area under curve - AUC 0.713). This study documented a response in 78% of patients with COVID-19 ARDS in the prone position, and this is similar to the 70% success rate reported in the literature. Factors associated with nonresponse were time from start of MV until prone positioning, the preprone PaO_2_/FIO_2_ value, and a mixed or multilobar-consolidation radiological pattern. Nonresponders also had higher mortality rates (54.1% *versus* 31.3%; p < 0.001).

Recognizing the factors associated with prone ventilation failure could help the identification of candidates for other rescue strategies, including more extensive prone positioning,^(8)^ PEEP titration (using electrical impedance or computerized thoracic tomography),^(9)^ inhaled nitric oxide and early veno-venous extracorporeal membrane oxygenation (ECMO).^(10)^
